# Genetic susceptibility in childhood acute leukaemias: a systematic review

**DOI:** 10.3332/ecancer.2015.539

**Published:** 2015-05-14

**Authors:** Gisele D Brisson, Liliane R Alves, Maria S Pombo-de-Oliveira

**Affiliations:** 1 Paediatric Haematology–Oncology Programme, Research Centre, Instituto Nacional de Câncer, Rio de Janeiro, Brazil, 20231050; 2 Pharmacy Service, Multiprofessional Residency Programme, Instituto Nacional de Câncer, Rio de Janeiro, Brazil, 20231050

**Keywords:** leukaemia, genetic polymorphism, genetic predisposition to disease, environmental exposure

## Abstract

Acute leukaemias (AL) correspond to 25–35% of all cancer cases in children. The aetiology is still sheltered, although several factors are implicated in causality of AL subtypes. Childhood acute leukaemias are associated with genetic syndromes (5%) and ionising radiation as risk factors. Somatic genomic alterations occur during fetal life and are initiating events to childhood leukaemia. Genetic susceptibility has been explored as a risk factor, since environmental exposure of the child to xenobiotics, direct or indirectly, can contribute to the accumulation of somatic mutations. Hence, a systematic review was conducted in order to understand the association between gene polymorphisms and childhood leukaemia risk. The search was performed in the electronic databases PubMed, Lilacs, and Scielo, selecting articles published between 1995 and 2013. This review included 90 case-control publications, which were classified into four groups: xenobiotic system (n = 50), DNA repair (n = 16), regulatory genes (n = 15), and genome wide association studies (GWAS) (n = 9). We observed that the most frequently investigated genes were: *NQO1, GSTM1, GSTT1, GSTP1, CYP1A1, NAT2, CYP2D6, CYP2E1, MDR1 (ABCB1), XRCC1, ARID5B*, and *IKZF1*. The collected evidence suggests that genetic polymorphisms in *CYP2E1, GSTM1, NQO1, NAT2, MDR1*, and *XRCC1* are capable of modulating leukaemia risk, mainly when associated with environmental exposures, such as domestic pesticides and insecticides, smoking, trihalomethanes, alcohol consumption, and x-rays. More recently, genome wide association studies identified significant associations between genetic polymorphisms in *ARID5B* e *IKZF1* and acute lymphoblastic leukaemia, but only a few studies have replicated these results until now. In conclusion, genetic susceptibility contributes to the risk of childhood leukaemia through the effects of gene–gene and gene–environment interactions.

## Introduction

### Childhood acute leukaemia

Acute leukaemias (AL) are the highest incidence malignancy in children and adolescents (≤19 years of age) and as a whole, the aetiology has not yet been unveiled. There are two major groups of AL, acute lymphoblastic leukaemia (ALL) and acute myeloid leukaemia (AML), classified according to characteristics presented by leukaemic cells, such as morphological features, surface antigens, chromosomal and molecular abnormalities [1], and gene expression profile [2].

Observational epidemiology has demonstrated that about 5 to 10% of AL are associated with ionising radiation exposure and congenital genetic syndromes (Down, neurofibromatosis, Fanconi anaemia, and Bloom syndrome) [3], that are associated with specific leukaemia subtypes. For the remaining 90% of AL, the aetiopathology is postulated to be a multistep process and somatic mutations are the start point of the pathway. When initiated during fetal life, the majority of them require postnatal events that contribute to accumulation of secondary mutations and proliferative advantage [3–5]. In this regard, the initiating event originating in cells would take advantage of the genetic predisposition conferred, in part, by genetic susceptibility and damage from exogenous exposures [6].

### Genetic susceptibility

Genetic polymorphisms are defined as natural genetic variations that occur randomly in the general population. The most common type is the single nucleotide polymorphism (SNP) that consists of a variation at a single base pair [7]. Depending on where it is located, SNPs can interfere with a gene’s function, affecting metabolic pathways [8]. This review will focus on three main pathways that have been related to AL genetic susceptibility: xenobiotic system, DNA repair system, and cell regulation, which have been identified as risk factors in childhood leukaemia.

### Xenobiotic system

Children are more vulnerable and susceptible to environmental toxicants than adults because of physiological immaturity, and also indirect and unintended exposures. Environmental agents such as tobacco and traffic smoke, pesticides, household chemicals, paintings, and diet are potential AL risk factors, as they may contain carcinogenic substances to humans, such as organic solvents (benzene derivatives), polycyclic aromatic hydrocarbons (PAHs), and organochloride compounds [9]. These substances, however, require metabolic activation by enzymes from the xenobiotic system to be able to interact with genetic material and eventually cause somatic mutations [10].

The xenobiotic system is classified into two phases: i) phase I enzymes, represented by cytochrome P450 isoenzymes (CYP), that catalyse hydrolysis, reduction, and oxidation reactions; and ii) phase II enzymes, that catalyse conjugation reactions, comprising glutathione S-transferases (GST) and N-acetyl transferases (NAT).

Interindividual genetic variations that are capable of altering the process of metabolism of pro-carcinogens, in both mother and child, may modulate the risk of developing paediatric leukaemia [10].

### DNA repair

DNA repair systems play an essential role in maintaining integrity and genomic stability [11]. Ionising radiation, environmental carcinogens and their reactive intermediates, together with genomic instability and inherent errors in DNA replication process contribute to the occurrence of damage in DNA. Mutations, chromosomal breaks, and crosslinks are actively recognised and repaired by sets of enzymes that constitute the DNA repair system [12]. Three main mechanisms are responsible for single-stranded DNA damage repair: i) base excision repair (BER), ii) nucleotide excision repair (NER), iii) and mismatch repair (MMR); which comprises enzymes enconded by several genes, such as *XRCC1, ERCC2, MLH1, MSH3*. Meanwhile, double-stranded DNA breaks can be repaired by homologous recombination (HR) or nonhomologous end joining (NHEJ), throughout enzymes as nibrin (NBN) and others, encoded by several genes, such as *ATM, BRCA2*, and *RAD51* [13]. Failures in those systems have been linked to birth defects, cancer, and premature ageing [12]. For instance, Fanconi anaemia, ataxia–telangiectasia, Nijmegen breakage syndrome, and Bloom syndrome, resulting from DNA repair disorders, are highly associated with childhood leukaemia [14].

### Regulatory genes

Cells often present signal transduction alterations that lead to proliferation in response to external factors. Several growth factors, their receptors and effector molecules have been identified as proto-oncogenes or tumour suppressor genes. Mutations in these genes may interfere with regulatory mechanisms that control cell cycle, leading to generation of malignant clones [15]. Considering this fact, polymorphisms in genes involved in cell cycle regulation also contribute to cancer susceptibility [16]. Nevertheless, few studies to date have investigated the association of regulatory genes with paediatric leukaemia.

Considering the large amount of epidemiologic data about this subject, it becomes necessary to use systematic methods to evaluate and synthesise all the information in order to facilitate communication between molecular epidemiology and clinical practice [17]. In an attempt to clarify some issues in this field, a systematic review was conducted which aimed to add comprehensive information about genetic susceptibility in childhood leukaemia.

## Methods

### Publication search strategy

A literature search on genetic susceptibility and childhood leukaemia was carried out using PubMed, Lilacs, and Scielo (last updated in June 2013). The following terms were used in different combinations: *‘acute lymphoblastic leukaemia’, ‘acute myeloid leukaemia’, ‘genetic polymorphism(s)’, ‘genetic susceptibility’, ‘xenobiotic(s)’, ‘molecular epidemiology’, ‘risk factor(s)’*, and ‘child* or infant or paediatric’. Also, the following MeSH terms were used: *‘precursor cell lymphoblastic leukaemia–lymphoma’, ‘leukaemia, myeloid, acute’, ‘polymorphism, genetic’, ‘genetic predisposition to disease’, ‘gene–environment interaction’, ‘case-control studies’*, and *‘genetic association studies’*. References were also checked in order to look for articles that were missing in the electronic databases. The search strategy was elaborated using the Preferred Reporting Items for Systematic reviews and Meta-Analyses (PRISMA) statement as a guideline [18].

### Inclusion and exclusion criteria

The inclusion criteria for all publications were: 1) case-control genotyping studies published between 1995 and 2013 that tested the risk of genetic polymorphisms with childhood ALL and/or AML (ages ≤ 21 years); 2) studies that provided sufficient data for estimating the risk association with odds ratio (OR), relative risk (RR) or interaction odds ratio (IOR); and 3) full text available in English, Spanish, and Portuguese. The exclusion criteria were: 1) studies with a different theme from what was proposed for this review; 2) publications in different languages, otherwise the ones specified; 3) studies that specifically include leukaemia cases related to genetic syndromes—Down, neurofibromatosis, Fanconi anaemia, Bloom syndrome and ataxia–telangiectasia—or secondary leukaemia; 4) articles that include other malignancies besides leukaemia in the same cohort, avoiding extrapolation of results exclusively for leukaemias; 5) articles about family gene transmissions; 6) articles about genes and prognosis; 7) articles about folate genes and immune system; and 8) comments and editorials.

### Data extraction

Information was extracted from each eligible article supervised by two investigators (LRA and MSPO), according to the inclusion criteria listed above. The following variables were collected from eligible studies: geographical origin, first author’s name, year of publication, leukaemia subtype, number of cases and controls, age, candidate genes investigated, and significant genotyping results.

### Statistical analysis

The strength of association between different genetic polymorphisms through the case-control method was evaluated by analysis of OR, RR, or IOR, with 95% confidence interval (95% CI), that were collected from the studies. Risk associations were considered significant when the P-value was ≤0.05.

## Results and discussion

**Figure 1.**
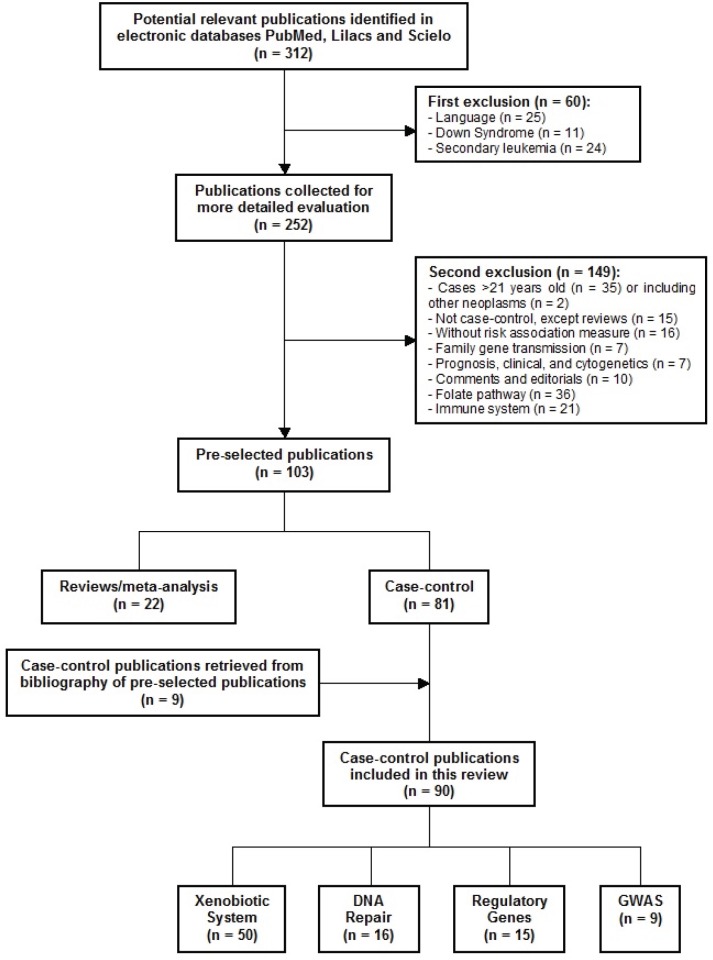
Flow diagram of included and excluded publications.

**Figure 2.**
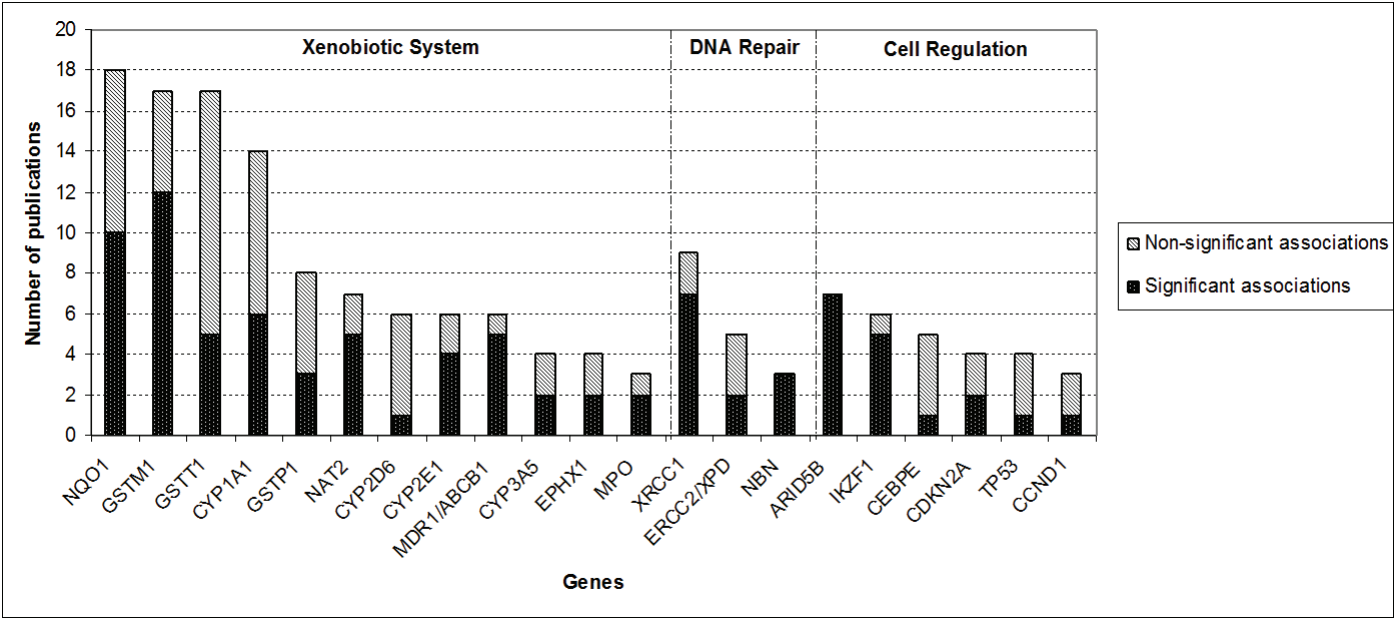
Proportion of publications that reported statistically significant associations between genetic polymorphisms and CAL risk for each of the genes that had polymorphism analysis reported by at least three publications.

**Figure 3.**
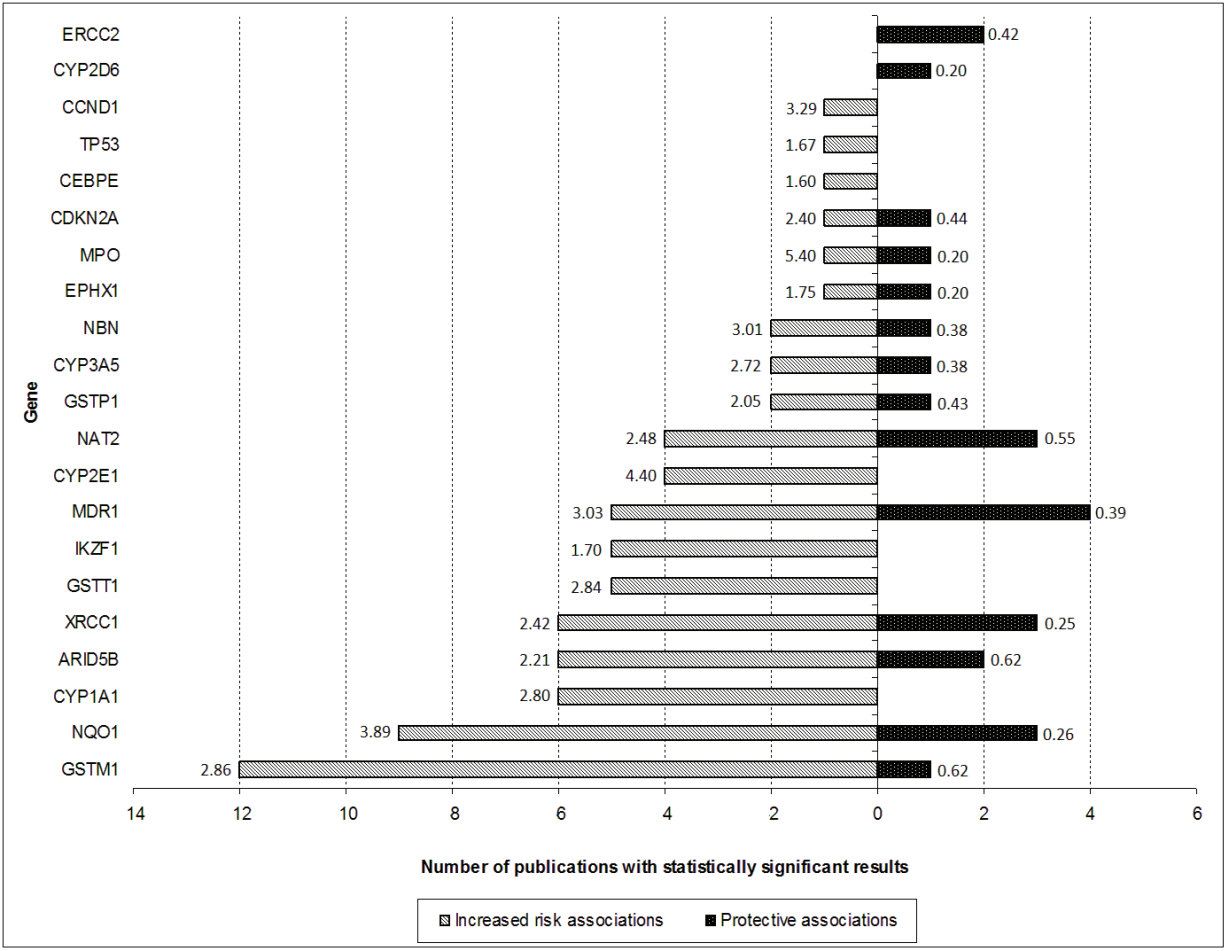
Number of publications that have shown statistically significant protective (black bar) or increased risk (white bar) associations for each gene (only genes with polymorphism analysis reported by at least three publications are shown). The numbers presented in the extremities of each bar represent the median ORs of risk associations found for polymorphisms involving each gene.

After screening of the retrieved titles, 312 publications were identified as potentially relevant to this review (Figure 1). After application of exclusion criteria, 103 publications were pre-selected, of which 22 were reviews, and 81 were case-control studies. After checking for bibliographies of pre-selected publications, nine papers were added, giving a total of 90 case-control publications included in this review. The majority of publications addressed polymorphisms in genes related to xenobiotic system (n = 50), followed by DNA repair genes (n = 16), regulatory genes (n = 15), and genome wide association studies (GWAS) (n = 9). The most frequently analysed gene polymorphisms, presented by at least three papers (Figure 2), were located in genes *CYP1A1, CYP2D6, CYP2E1, CYP3A5, EPHX1, GSTM1, GSTT1, GSTP1, MPO, NAT2, NQO1, MDR1 (ABCB1), XRCC1, ERCC2, NBN, ARID5B*, and *IKZF1*. Figure 2 also shows the proportion of publications that showed statistically significant associations for each gene, among the total. Considering only statistically significant results, median ORs for increased risk or protective associations for those genes were calculated, and are demonstrated in Figure 3.

### Xenobiotic system

Genetic susceptibility studies related to xenobiotic system are presented in Table 1. The main investigated gene polymorphisms comprised the genes *CYP1A1, CYP2D6, CYP2E1, CYP3A4, CYP3A5, EPHX1, GSTM1, GSTP1, GSTT1, MDR1, MPO, NAT1, NAT2*, and *NQO1*. Most publications are from Asia (39.6%), followed by North America (25%), Europe (20.8%), and South America (14.6%). The great disparity between ethnic groups is remarkable, since the Asian continent includes a wide range of people with distinct genetic backgrounds, such as Caucasians, Turkish, Indians, Japanese, Chinese, and the Korean population, likewise, Americans have diverse ancestries, mainly Caucasian, Hispanic, and African. The vast majority of publications (75%) investigated the genetic susceptibility in ALL only, and 25% in AML + ALL; no one has investigated AML solely.

Regarding phase I metabolism, fourteen publications [19–32] explored polymorphisms of *CYP1A1*, mainly the variant alleles *2A, *2B, *2C, and *4; only six (42.9%) found significant associations between these alleles and childhood ALL. CYP1A1*2A allele was associated with increased risk for ALL among Canadians [27], Indians [26], and Hispanics in North-America [31]. CYP1A1*2B was associated with increased risk for B-cell precursor ALL (Bcp-ALL) among north-Americans [31]. CYP1A1*2C allele was also associated with increased risk for ALL among Indians [26] and north-Americans [31]. Two Brazilian papers found significant associations of *CYP1A1* variant alleles and ALL only with combined genotypes: CYP1A1*2 + CYP2E1*5B + GSTP1*B + *GSTM1*-null [23], and CYP1A1*2A/*2B/*2C + *NQO1* 609-CT/ CT + TT [32].

Six publications [19, 22–24, 33, 34] explored variant alleles of *CYP2E1*, mainly *5B, *6 and *7B; in four out of six (66.7%), significant associations were found: CYP2E1*5B was related to increased risk for ALL/AML in Canadians [33] and Turkish [19]. The presence of at least two variant alleles (*5B and *6; *6 and *7B; or *5B, *6, and *7B) was related to increased risk for ALL in Turkish [34]. Besides, the combined genotype CYP2E1*5B + CYP1A1*2 + GSTP1*B + *GSTM1*-null was associated with increased risk for ALL in Brazilians [23].

Six publications [19, 24–27, 35] explored CYP2D6*3 and *4 alleles. The wild-type allele (CYP2D6*1) was negatively associated with ALL in Brazilians [35]. Polymorphisms of *CYP3A* genes (CYP3A4*1B, CYP3A5*3, and CYP3A5*6) were investigated in four papers [24, 29, 36, 37], and no significant associations were found for CYP3A4*1B allele [24, 29]. The wild-type allele CYP3A5*1 was associated with ALL increased risk in Denmark and Norway [36], whereas alleles CYP3A5*3 and CYP3A5*6 were associated with ALL protection in Brazilians [37]. The same Brazilian study found increased risk association for the CYP3A5*6 allele only in Whites, leading to speculation that *CYP3A5* may also be involved in detoxification as well as activation mechanisms of carcinogens. Alleles that do not produce a functional protein, such as CYP3A5*6, would contribute to accumulation of potentially harmful substances [36].

Eighteen publications [21, 22, 24, 25, 32, 33, 35, 38–48] addressed polymorphisms of *NQO1* gene (C609T and C465T). In ten studies (55.5%), divergent associations were found. *NQO1* 609T allele was associated with increased risk for ALL among Canadians [33] and Brazilians [32]. In Filipinos, the genotype *NQO1* 609CC was associated with increased risk for ALL [44], whereas the variant allele *NQO1* 609T was negatively associated with ALL in Malaysian boys [48] and Brazilians [35]. The 609T allele has been associated with infant leukaemia and MLL gene rearrangements in Caucasians [39, 42, 46, 47]. Otherwise, very few publications demonstrated increased risk for ALL in Canadians [33], and Japanese [40] with *NQO1* 465T allele.

Few publications [22, 24, 35, 49] have explored polymorphisms of EPHX1*2 (T28C–Tyr113His) and EPHX1*3 (A52G–His139Arg); two out of four showed divergent results: variant alleles *2 and *3 were associated with protection for ALL in Brazilians [35], whereas the *2 allele was associated with increased risk for ALL in Turkish [49]. Given the duality of functions that enzyme *EPHX1* performs, its interconnection with CYP450 family, the diversity of xenobiotics presented in the environment, and the differences in allele frequencies among populations, gene polymorphisms of *EPHX1* may contribute in an unpredictable way in activation or detoxification of xenobiotics.

Finally, three publications [24, 33, 35] demonstrated that the variant alleles of *MPO*, mainly MPO*2 (G-463A) have a protective effect when combined to other gene polymorphisms [33, 35]. No study has ever demonstrated an independent effect of MPO*2 in childhood leukaemia susceptibility.

Regarding phase II metabolism, the *GST* gene family was the most investigated (20 case-control publications), regarding association with childhood leukaemia risk all over the world. In twelve out of seventeen [19, 20, 23–27, 29, 38, 44, 48, 50–55] the homozygous deletion of *GSTM1* and *GSTT1* alleles (null genotype) was related to childhood leukaemia risk: *GSTM1*-null genotype was associated with increased risk for ALL in ten papers [23, 26, 27, 29, 38, 44, 50, 52, 55]; *GSTT1*-null genotype was associated with increased risk for ALL, mostly when combined with *GSTM1*-null genotype [29, 38, 52, 55]. However, two studies performed in US children disclosed conflicting results: *GSTM1*-null was found to be associated with protection for ALL among non-Hispanic American children [24], whereas non-null alleles GSTM1*A, GSTM1*B and GSTT1*1 were associated with increased risk for ALL [51]. These discrepancies may be because of differences in allele frequencies in mixed populations and the different patterns of environmental exposures.

From eight publications [20, 23, 24, 38, 51, 54, 56, 57] that explored *GSTP1* polymorphisms (A1578G–GSTP1*B; C2293T–GSTP1*C), only three (37.5%) showed significant associations, but with opposite results: GSTP1*B was related to increased risk for ALL among Canadians [57] and Brazilians [23], while a protective effect was observed for Indonesian girls [38]. Seven publications [22, 24, 25, 37, 58–60] analysed the complexity of *NAT2* polymorphisms. Overall, haplotypes that result in low activity phenotype were associated with increased risk for childhood leukaemia [59]. The only exception was a protective effect of *NAT2* 341C-481T-590A, that results in slow activity phenotype, among Russians [25]. Besides, the combination of slow phenotype in both child and mother intensified the risk for early age ALL [60]. The rapid allele *4 was associated with protection for ALL among Canadians [58], as well as for AML in Brazilian children [59].

Besides the detoxifying function of some metabolising enzymes, membrane transporter proteins also act protectively against carcinogens. Six publications [24, 48, 61–64] explored polymorphisms of *MDR1* (*ABCB1* family) gene, mainly C1236T, G2677T/A, C3435T, and T-129C, that encodes an efflux membrane transporter (P-glycoprotein) with childhood ALL. *MDR1* 3435T allele was consensually associated with increased risk for ALL in four publications [61–64], while the haplotype GAGT (rs2520464, rs12334183, rs1202179, rs17327442) was associated with protection for ALL in north-Americans [24].

Finally, few publications have explored other genes, such as *ARNT, CYP1A2, CYP1B1, CYP2C8* and *IDH1* [24], *PON1* [39], *NAT1* [58], *GSTO1*, and *GSTO2* [65], and *AKR1C3* [66], in childhood ALL. Their results, however, need to be replicated in further studies.

### DNA repair

The data from genotyping studies in genes related to DNA repair system are summarised in Table 2. The main investigated gene polymorphisms comprised the genes *ERCC2, MLH1, MSH3, NBN*, and *XRCC1*. Six publications (37.5%) were performed in Europeans, five (31.25%) in Asians, four (25%) in north-Americans, and one (6.25%) in Brazilians. The majority of them focused only in ALL.

Nine case-control publications [67–75] addressed polymorphisms of *XRCC1* (Arg194Trp, Arg280His, and Arg399Gln), which encodes a protein involved in BER pathway. Seven of them (77.8%) have shown significant associations with childhood ALL. *XRCC1* 194Trp allele was related to increased risk among Turkish girls [67], Indians [71], and Mexicans [72], while it was related to protection among Thai [73]. No publication showed association of 280His allele with childhood ALL. *XRCC1* 399Gln allele was related to increased risk for ALL among Indians [71], Thai [73], Turkish [75], and Poles [74]. However, when combined with *XRCC1* 194Arg wild-type allele and the variant alleles *ERCC2* 751Gln and *TYMS* 3R, the *XRCC1* 399Gln allele was related to protection for ALL among Brazilians [68].

The *ERCC2* gene encodes a DNA helicase involved in NER pathway, and its polymorphisms (Asp312Asn and Lys751Gln), were explored in five case-control publications [67–70, 73]. No one was able to show an independent association with leukaemia risk. *ERCC2* 751Gln allele was related to protection for ALL among Brazilians when combined with *XRCC1* 399Gln-194Trp and *TYMS* 3R [68]. The haplotype GAA (rs3916874, rs238416, rs171140) was also associated with protection for ALL among north-Americans [70].

Genetic polymorphisms of *NBN*, which is involved in DNA repair by HR, were reported by three publications [70, 76, 77]. Two of them showed that 657del5 mutation was related to increased risk for ALL among Poles [76, 77]. Recently, five SNPs of *NBN* gene (rs12680687, rs6470522, rs7840099, rs1805812, rs709816) were associated with protection for a subset of Bcp-ALL in north-Americans [70], reinforcing that the interaction of multiple polymorphisms can influence paediatric leukaemia risk.

Polymorphisms of two genes involved in MMR, *MLH1*, and *MSH3*, were explored in two papers [21, 78], but both failed to demonstrate any independent association with childhood ALL. However, the combination of *MLH1* 219-Ile/Ile with genetic variants of *CYP2E1* or *GSTM1* and *CYP1A1* increased the risk for ALL [78]. Other genotyping studies of *OGG1* [74, 79], *MUTYH* [74], *ERCC1* [80], *XRCC4* [70, 81], *APEX1*, *BRCA2*, and *RAD51* [70] found increased associations with childhood ALL.

### Regulatory genes and GWAS

To date, the majority of publications regarding regulatory genes and childhood leukaemia were performed to validate GWAS results. The main investigated gene polymorphisms are summarised in Table 3. The majority of publications focused only in ALL (87.5%) and is from North America, followed by Asians (25%), and Europeans (25%).

Prior to GWAS publications, a few genetic polymorphisms of regulatory genes were investigated by candidate gene approach. *CCND1* 870AA genotype (homozygous wild-type) was related to increased risk for ALL among Chinese [82]. Polymorphisms in promoter regions of genes *CDKN2A* (T-222A), *CDKN2B* (C-1270T, A-593T, C-287G) and *CDKN1B* (G-1608A) were also associated with childhood pre-B ALL risk among Canadians [16]. Furthermore, the Arg72Pro polymorphism in *TP53* gene was associated with increased risk for ALL among children from the United Kingdom [83]. Polymorphisms of other genes were also related to childhood acute leukaemia (CAL) risk, like *BAT3* and *DAXX* [83], *ATM* [84], *TERT* [85], *MDM2* [86], *LMO1* [21], *MLL* and *EP300* [87], but the associations need to be replicated in further studies.

The first two GWAS regarding genetic susceptibility to childhood leukaemia were published in 2009, which observed that SNPs in regions 7p12.2 (*IKZF1* rs4132601, rs11978267), 10q21.2 (*ARID5B* rs7089424, rs10821936, rs10994982), and 14q11.2 (*CEBPE* rs2239633) were associated with childhood ALL risk, specifically with B-cell acute lymphoblastic leukaemia (B-ALL), and hyperdiploid subsets, with ORs ranging from 1.34 to 1.91, and P <10–7 [88, 89]. In subsequent analysis, the association of *CDKN2A* rs3731217 (T allele) with protection for ALL (OR 0.71, P = 3.01 x 10–11) was validated [90], and *ARID5B* rs10821936 was associated with ALL among Blacks (OR 2.08, P = 0.0015), mainly hyperdiploid B-ALL (OR 6.62, P <0.001) in the US [91]. Also, it was demonstrated that 24% of the total variation in B-cell precursor acute lymphoblastic leukaemia (Bcp-ALL) risk is accounted for common genetic variation, which supports for a polygenic basis for susceptibility to Bcp-ALL [92]. French studies have shown similar associations regarding *IKZF1* and *ARID5B*, and also found associations between *CDKN2A* rs3731217 (OR 0.8) and *CEBPE* rs2239633 (OR 0.9) with ALL risk [93].

Since then, other research groups have aimed to replicate the risk associations previously identified by the GWAS. As shown in Table 3, seven papers confirmed *ARID5B* rs10821936 association with increased ALL risk, mainly B-ALL [43, 77, 94–98]. From six publications that explored *IKZF1* SNPs [21, 43, 77, 95–97], five of them confirmed the risk associations for ALL. *CDKN2A* and *CEBPE* SNPs associations were replicated by one study respectively [70, 95].

SNPs in other genes were also identified as risk variants for ALL by other GWAS, but replication of these results are still needed: *HAO1* (rs6140264, OR 8.84), *EPB41L2* (rs9388856, rs9388857, rs1360756, OR 8.97), *C2orf3* (rs12105972, OR 0.13), and *MAN2A1* (rs3776932, OR 0.11) were associated with ALL risk among Koreans (P = 0.0001) [99]; *TP63* (rs17505102, OR 0.63, P = 4.87 x 10–7), *PTPRJ* (rs3942852, OR 0.77, P = 2.54 x 10–4), and *EPOR* (rs4804164, OR 0.58, P = 0.008; rs317913, OR 0.60, P = 0.019) were associated with *ETV6/RUNX1* ALL risk among Europeans [100, 101].

### Gene-environment interaction

Few publications addressed the interaction between gene polymorphisms, environmental exposures, and childhood leukaemia. Studies performed in children exposed directly and/or indirectly throughout maternal exposures are scarce. To date, environmental exposures that have been explored were smoking (tobacco exposure), pesticides, insecticides, trihalomethanes (chlorination by-products of drinking water) alcohol consumption, paint use and x-rays. The absence of *CYP1A1* CGACC haplotype, consisting of five SNPs (-T1761C, -G9893A, Ex7+A131G, C1188T, C11599G), was associated with increased risk for ALL among children with father’s smoking or at least one smoker at home [28]. Also, haplotypes of *CYP2C8* and *MDR1* (*ABCB1*) were related to increased risk for ALL when associated with paint use and indoor insecticides, respectively among Hispanics living in the US [24]. Another finding was that children exposed to indoor insecticides carrying *MDR1* haplotype CGC (C1236T, G2677T/A, C3435T) presented a lower risk for ALL [64], indicating that pesticides’ toxic effects may be influenced by efflux through P-glycoprotein complex.

Six publications [102–107] estimated risk associations by calculating IOR in a case-only cohort. It was observed that CYP1A1*2A/*2B increased the risk for ALL by five-fold among children exposed to pesticides during maternal pregnancy and childhood, while CYP1A1*2B was related to a protective effect among children with mothers who had smoked 1–20 cigarettes during the first trimester of pregnancy (IOR = 0.1; IC95% 0.01–0.9) and with fathers who had smoked >20 cigarettes between birth and diagnosis (IOR 0.2; IC95% 0.04–0.9) [102, 103]. Also, it was observed that among children exposed to higher levels of trihalomethanes in drinking water, the risk for ALL was increased in the presence of the polymorphic variant CYP2E1*5 (IOR 9.75; IC95% 1.10–86.01), and *GSTT1* deletion (IOR 9.13; IC95% 1.44–57.82), in pre and post-natal periods, respectively [104]. *GSTM1*-null genotype and CYP2E1*5 variant were related to increased risk for ALL among children with mothers who had consumed alcoholic beverages during the third trimester of pregnancy (IOR 2.4; IC95% 1.1–5.4) and nursing period (IOR 4.9; IC95% 1.4–16.6), respectively [105]. Thus, it is notable that variations in xenobiotic metabolism resulted from genetic polymorphisms can modulate childhood leukaemia risk.

Concerning DNA repair genes, it was observed that variants of *APEX1* (Asp148Glu) and *MLH1* (Ile219Val) were associated with a protective effect for ALL among girls exposed to x-rays (one or more exposures) during postnatal period (*MLH1* IOR 0.2; IC95% 0.1–0.8; and *APEX1* IOR 0.1; IC95% 0.0–0.7) [106, 107]. Also, the protective effect for ALL of *XRCC4* GGG haplotype, consisting of SNPs rs1193695, rs301276, and rs301287, was modulated by number of postnatal x-rays (P = 0.027) [70]. Again, cancer susceptibility resulted from the interaction of environmental exposure and genetic polymorphism, which highlights the multifactorial aetiology of paediatric leukaemia.

## Conclusions

Great scientific advances in the understanding of paediatric leukaemia have been made. Unlike the adult, who usually develops cancer because of the cumulative effect of environmental exposures during his life, the child, which manifests leukaemia with a short latency period, does not have enough exposure time to allow the initiation of a long carcinogenic process. Thus, genetic susceptibility may play an important role in modulating environmental exposures’ effects.

This systematic review gathered publications up to 2013 and was an attempt to overview the risk associations between several gene polymorphisms and paediatric AL. It was possible to collect from the selected studies significant amount of data, which is considered to be a fair representation of international scientific literature on this subject. The vast majority of studies so far focused on evaluating the magnitude of risk of genetic polymorphisms in ALL, mainly because Bcp-ALL is the most frequent type of leukaemia in children. In this context, we also realised that there is still a great need for further investigations on the risk factors for paediatric AML.

Regarding the xenobiotic system, gene polymorphisms of *CYP2E1, GSTM1, NQO1, NAT2*, and *ABCB1* (*MDR1*) were more frequently associated with childhood leukaemia risk, which also showed interaction effect with environmental exposures such as paints, household pesticides, insecticides, smoking, alcohol, and trihalomethanes. Gene polymorphisms related to DNA repair have been little investigated in paediatric leukaemia, maybe because of its association with genetic diseases. However, it was noticed that *XRCC1* polymorphisms play an important role in the development of ALL, and postnatal exposure to x-rays can modulate leukaemia risk in the presence of *APEX1*, *MLH1*, and *XRCC4* gene variants. While interpreting these results, one has to consider that fetuses and infants are naturally more affected than adults by a variety of environmental toxicants, mainly because of differential exposure and physiologic immaturity, which makes them more susceptible to suffer from DNA damage and less capable of detoxifying carcinogenic compounds [108]. And so, genetic polymorphisms involving xenobiotic and DNA repair systems have a major role in modulating the effects of environmental agents in children.

Some limitations were observed in the studies that might make the consolidation of scientific evidence difficult, such as: 1) relatively small number of cases, making it difficult to obtain statistically significant results; 2) ethnic and racial differences between populations, which are reflected in distinct polymorphic allele frequencies and patterns of exposure to environmental agents; 3) little information regarding the effect of gene polymorphisms on the encoded protein; 4) and few considerations about gene–environment interactions. Also, we could observe that the majority of associations provided low risk estimates (OR <2.0), which showed that gene polymorphisms are of low penetrance, and conceptually, are minor parts of multifactorial pathways to childhood leukaemia [6].

More recently, GWAS have identified new gene polymorphisms potentially related to paediatric ALL, particularly involving *IKZF1* and *ARID5B*, which were subsequently replicated in independent studies. As a result, we see genetic susceptibility clearly contributes to childhood leukaemia risk, mainly through gene–gene and gene–environment interactions. Further studies are still needed to confirm the observed associations in different populations and to characterise environmental agents as risk factors for childhood leukaemia.

## Conflicts of interest

The authors declare no conflict of interest.

## Authors’ contributions

The study was designed by GDB, LRA, and MSPO. The literature search and data analysis were carried out by GDB, with the supervision of LRA and MSPO. The manuscript was prepared by GDB, LRA, and MSPO.

## Figures and Tables

**Table 1.** Genetic susceptibility publications in childhood leukaemia involving genes related to xenobiotic system.

Continent	Country	Leukaemia subtype	Number of cases/controls	Ages of cases (years)	Investigated genes	Significant results			First author, year	Reference
						Genetic variation	OR (95% CI)	P-value		
**Europe**	United Kingdom	ALL, AML	36/100	<15	*NQO1*	*NQO1* 609-T in AL *MLL+* *NQO1* 609-T in AL *MLL/AF4*	2.54 (1.08–5.96) 8.63 (2.45–33.22)	0.015 <0.001	Wiemels, 1999	[47]
	Portugal	ALL	47/102	≤18	*GSTM1, GSTT1*	*GSTM1*-null	2.20 (1.10–4.50)	0.035	Alves, 2002	[50]
	Poland	ALL	113/175	≤18	*MDR1*	*MDR1* 3435-TT	1.80 (1.10–3.10)	0.037	Jamroziak, 2004	[62]
	Germany/Austria/Czech Republic	ALL	209/190	≤19	*NQO1*	No significant associations were found.	–	–	Kracht, 2004	[41]
	Italy	ALL	156/147	<15	*NQO1*	*NQO1* 609-CT+TT in AL *MLL*- ≤12 months	4.22 (1.43–12.49)	0.006	Lanciotti, 2005	[42]
	Italy	ALL	323/384	<18	*GSTM1, GSTT1, GSTP1*	No significant associations were found.	–	–	Pigullo, 2007	[54]
	Hungary	ALL	396/192	1–15	*MDR1, BCRP*	*MDR1* 2677G-3435T haplotype *MDR1* 2677T-3435C haplotype	2.50 (1.40–4.40) 0.40 (0.20–0.80)	0.002 0.006	Semsei, 2008	[63]
	Hungary	ALL	543/529	1–15	*AhR, NQO1, NQO2*	No significant associations were found.	–	–	Lautner-Csorba, 2012	[43]
	Denmark/Norway	ALL	616/203	1–15	*CYP3A5*	CYP3A5*3 (6986-A allele)	1.64 (1.01–2.66)	0.049	Borst, 2011	[36]
	France	ALL, AML	493/549	<15	*CYP1A1, CYP2E1, NQO1, NAT2, EPHX1*	NAT2*5 in ALL	1.80 (1.30–2.50)	NA	Bonaventure, 2012	[22]
**Asia**	Turkey	ALL, AML	177/185	≤17	*GSTM1, GSTT1, GSTP1, CYP1A1*	No significant associations were found.	–	–	Balta, 2003	[20]
	Turkey	ALL, AML	273/286	1–16	*NQO1*	No significant associations were found.	–	–	Sirma, 2004	[45]
	Turkey	ALL, AML	163/140	2–18	*GSTM1, GSTT1, CYP1A1, CYP2D6, CYP2E1*	CYP2E1*5B (–/+) in ALL CYP2E1*5B (–/+) in AML	3.40 (1.30–9.10) 4.90 (1.60–15.20)	0.010 0.006	Aydin-Sayitoglu, 2006	[19]
	Turkey	ALL	168/207	1.5–15.5	*CYP2E1*	Co presence of at least 2 variant *CYP2E1* alleles (*5B and *6; *6 and *7B; *5B, *6 and *7B)	3.90 (1.40–11.00)	<0.050	Ulusoy, 2007	[34]
	Turkey	ALL	167/190	1.5–15.5	*EPHX1*	*EPHX1* 113-His (28T>C) *EPHX1* 113-His (28T>C) + *XRCC1* 399-Gln	1.40 (1.00–2.00) 2.10 (NA)	0.030 0.030	Tumer, 2012	[49]
	India (South)	ALL	118/118	≤14	*GSTM1, GSTT1, CYP1A1, CYP2D6*	CYP1A1*2A (+/+) CYP1A1*2A (–/+) CYP1A1*2C (+/+) CYP1A1*2C (–/+) *GSTM1*-null	6.22 (1.30–29.71) 2.58 (1.41–4.72) 4.28 (1.14–16.11) 2.18 (1.16–4.10) 2.10 (1.21–3.67)	0.022 0.002 0.032 0.015 0.009	Joseph, 2004	[26]
	Japan	ALL, AML	103/197	<1.5	*NQO1*	*NQO1* 465-CT/TT in ALL *MLL+* *NQO1* 465-CT/TT in ALL *MLL-AF4*	3.55 (1.13–11.10) 6.36 (1.84–21.90)	0.020	Eguchi-Ishimae, 2005	[40]
	Japan	ALL	157/96	1–15	*MDR1*	*MDR1* -2352A in children ≥ 6 years of age *MDR1* 3435T	0.34 (0.20–0.77) 1.61 (1.09–2.39)	0.012 0.020	Hattori, 2007	[61]
	Thailand	ALL	107/320	≤14	*GSTM1, GSTT1, CYP1A1, CYP3A4, CYP3A5*	*GSTM1*-null *GSTM1*-null + *GSTT1*-null	1.70 (1.00–2.70) 1.70 (1.10–2.90)	0.040 0.020	Pakakasama, 2005	[29]
	Thailand	ALL	100/100	≤14	*GSTP1*	No significant associations were found.	–	–	Gatedee, 2007	[56]
	Thailand	ALL	99/100	1–14	*GSTO1, GSTO2*	GSTO1*140A/D GSTO2*142N/D in high risk ALL	2.24 (1.16–4.35) 5.52 (1.72–17.71)	0.009 0.004	Pongstaporn, 2009	[65]
	Russia	ALL, AML	403/490	≤17	*GSTM1, GSTT1, CYP1A1, CYP2D6, CYP2C9, CYP2C19, NQO1, NAT2*	*GSTT1*-null+*GSTM1*-null *NAT2* 341C+C481T+G590A	3.09 (2.05–4.65) 0.55 (0.33–0.93)	<0.001 0.026	Gra, 2008	[25]
	Philippines	ALL	60/60	<18	*GSTM1, GSTT1, NQO1*	*GSTM1*-null *NQO1* 609-CC *GSTM1*-null + *NQO1* 609-CC	2.37 (1.11–5.04) 4.82 (2.18–10.60) 11.9 (3.45–41.09)	0.020 <0.001 NA	Rimando, 2008	[44]
	Taiwan	ALL, AML	114/220	<20	*AKR1C3*	rs10508293 A > G in the child rs10508293 A > G in both child and mother	2.46 (1.69–3.58) 1.63 (1.30–2.04)	<0.001 <0.001	Liu, 2008	[66]
	Korea	ALL, AML	176/298	≤18	*CYP1A1*	Absence of haplotype *CYP1A1* CGACC (-T1761C, -G9893A,Ex7+A131G, C1188T, C11599G) in children with father’s smoking or at least one smoker at home, respectively (risk for ALL)	2.80 (1.50–5.30) 2.30 (1.20–4.40)	0.030 0.020	Lee, 2009	[28]
	China	ALL	67/146	0.8–18	*GSTM1, GSTT1*	*GSTM1*-null *GSTM1-T1*-null	2.86 (1.49–5.46) 3.15 (1.71–5.79)	<0.001 <0.001	Wang, 2004	[55]
	China/Malaysia	ALL	756/756	*med.* 4.8	*GSTM1, GSTT1, NQO1, MDR1*	*NQO1* 609-CT in Malay boys	0.38 (0.22–0.66)	0.001	Yeoh, 2010	[48]
	Indonesia (Javanese children)	ALL	185/177	≤14	*GSTM1, GSTT1, GSTP1, NQO1*	*GSTM1*-null in boys *GSTT1*-null in girls GSTP1*B in girls	1.89 (1.04–3.44) 2.20 (1.10–4.37) 0.43 (0.21–0.89)	0.050 0.027 0.031	Chan, 2011	[38]
	Iran	ALL	85/94	<16	*CYP1A1*	No significant associations were found.	–	–	Razmkhah, 2011	[30]
**North America**	Canada	ALL	177/304	1–21	*GSTM1, GSTT1, CYP1A1, CYP2D6*	*GSTM1*-null CYP1A1*2A (+/+, +/–) *GSTM1*-null + CYP1A1*2A (+/+, +/–)	1.80 (1.20–2.60) 1.80 (1.10–3.10) 3.30 (1.60–6.90)	0.004 0.030 0.002	Krajinovic,1999	[27]
	Canada	ALL	176/306	*med.* 6.0	*NAT1, NAT2*	NAT2*4 allele NAT2*5C allele NAT2*7B allele NAT1*4/*4 + *NAT2*-slow	0.60 (0.50–0.90) 3.10 (1.10–8.50) 2.90 (1.10–7.40) 1.90 (1.10–3.40)	0.010 0.020 0.030 0.030	Krajinovic,2000	[58]
	Canada	ALL	174/337	*med.* 5.2	*NQO1, CYP2E1, MPO*	CYP2E1*5B (–/+) NQO1*2 (C609T) or *3 (C465T) CYP2E1*5B (–/+) + NQO1*2/*3 + MPO*2 (–/–)	2.80 (1.20–6.70) 1.70 (1.20–2.40) 5.40 (1.20–23.40)	0.020 0.008 0.003	Krajinovic, 2002	[33]
	Canada	ALL	278/302	*med.* 4.9	*GSTP1*	GSTP1*A/B; B/B GSTP1*A/B; B/B in girls GSTP1*A/B; B/B + *GSTM1* null	1.50 (1.10–2.00) 1.90 (1.20–3.10) 2.20 (1.30–3.50)	0.020 0.010 0.002	Krajinovic, 2002	[57]
	United States	ALL	197/416	≤18	*GSTM1, GSTT1*	*GSTM1*-null + *GSTT1*-null in Blacks	7.36 (2.61^a^)	<0.001	Chen, 1997	[52]
	United States	ALL, AML	39/56	≤18	*NQO1*	*NQO1* 609-CT/TT in AL *MLL+* *NQO1* 609-CT/TT in ALL*MLL+*	2.47 (1.08–5.68) 3.35 (1.13–9.82)	0.033 0.028	Smith, 2002	[46]
	United States	ALL	171/NA	≤18	*GSTM1, GSTM3, GSTT1, GSTP1*	GSTM1*A GSTM1*B GSTT1*1	5.66^b^ (2.58–12.41) 4.28 ^b^ (1.79–10.19) 2.59 ^b^ (1.07–6.29)	<0.001 0.001 0.035	Barnette, 2004	[51]
	United States	ALL	76/76	≤6	*GSTM1, GSTT1*	No significant associations were found.	–	–	Klotz, 2006	[53]
	United States	ALL	294/369	<15	*MDR1*	*MDR1* 1236-TT *MDR1* 2677-TA/TT/AA *MDR1* 3435-TT in non-Hispanic White hyperdiploid ALL *MDR1* haplotype CGC(C1236T, G2677T/A, C3435T) x indoor insecticide exposure	40.35 (3.00–542.60) 6.01 (1.12–32.23) 8.86 (1.35–58.03) 0.37 (0.15–0.88)	NA NA NA 0.025	Urayama, 2007	[64]
	United States	ALL	163/251	<21	*CYP1A1, NQO1*	No significant associations were found.	–	–	Beuten, 2011	[21]
	United States	B-ALL	258/646	*med.* 6.3	*CYP1A1*	CYP1A1*2C (+/+) CYP1A1*2B (+/+) rs4886605 (–/+; +/+) in Caucasians CYP1A1*2A (+/+) in Hispanics CYP1A1*2B (+/+) in Hispanics CYP1A1*2C (+/+) in Hispanics	2.51 (1.18–5.33) 3.24 (1.43–7.34) 1.58 (1.01–2.46) 2.70 (1.27–5.74) 3.28 (1.40–7.69) 2.47 (1.13–5.38)	0.016 0.005 0.043 0.010 0.006 0.023	Swinney, 2011	[31]
	United States	ALL	377/448	<14	250 SNPs in42 genes^c^ + *GSTM1, GSTT1*	*MDR1* haplotype *ARNT* haplotype *CYP2C8* haplotype *CYP1A2* haplotype in non-Hispanics *CYP1B1* haplotype in non-Hispanics *GSTM1*-null in non-Hispanics *IDH1* haplotype in Hispanics *GSTM1*-null in Hispanics *CYP2C8* haplotype in Hispanics associated with paint use *MDR1* haplotype in Hispanics associated with indoor insecticides	0.44 (0.23–0.85) 4.93 (1.94–12.53) 3.18 (1.45–6.95) 2.19 (1.28–3.77) 0.11 (0.02–0.56) 0.62 (0.43–0.89) 6.12 (1.75–21.36) 1.85 (1.19–2.88) 1.67 (1.21–2.30) 3.03 (1.59–5.78)	0.015 0.001 0.004 0.005 0.007 0.010 0.005 0.007 0.001 0.005	Chokkalingam, 2012	[24]
**South America**	Brazil	ALL	113/221	≤18	*GSTM1, GSTT1, GSTP1, CYP1A1, CYP2E1*	GSTP1*B + *GSTM1*-null + CYP1A1*2 + CYP2E1*5B	10.30 (1.00–111.80)	0.050	Canalle, 2004	[23]
	Brazil	ALL	99/99	*med.* 4.0	*CYP1A1, NQO1*	*NQO1* 609-CT+TT *NQO1* 609-CT+TT CYP1A1 *2A/*2B/*2C	2.64 (1.46–4.80) 10.71 (1.20–95.46)	0.001 0.030	Yamaguti, 2010	[32]
	Brazil	ALL	206/364	<18	*CYP2D6,EPHX1,MPO,NQO1*	EPHX1*2 (–/+; +/+) CYP2D6*1 + EPHX1*2 + MPO*2 + NQO1*1 CYP2D6*1 + EPHX1*2 + MPO*1 + NQO1*2 CYP2D6*1 + EPHX1*2 + MPO*2 + NQO1*2 CYP2D6*1 + EPHX1*2 and *3 + MPO*2 + NQO1*2	0.26 (0.16–0.42) 0.20 (0.05–0.70) 0.46 (0.04–0.60) 0.06 (0.01–0.40) 0.20 (0.04–0.90)	0.001 0.003 0.001 <0.001 0.010	Silveira, 2010	[35]
	Brazil	ALL	132/131	≤1.75	*NAT2*	NAT2*5 NAT2*6 NAT2*5/*6 (slow) NAT2*5/*7 (slow) NAT2*5/*14 (slow) *NAT2* slow in both child andmother	2.41 (1.23–4.78) 2.32 (1.13–4.80) 11.70 (2.00–118.40) 6.95 (1.08–74.20) 13.50 (1.37–174.20) 30.00 (5.870–279.70)	NA NA NA NA NA NA	Zanrosso, 2010	[60]
	Brazil	ALL, AML	232/303	≤10	*NAT2*	*NAT2* 341-C in ALL *NAT2* 341-C in AML *NAT2* 590-A in AML *NAT2* slow phenotypes *NAT2* rapid phenotypes	2.30 (1.51–3.51) 2.48 (1.38–4.51) 1.57 (1.07–2.30) 2.42 (1.71–3.44) 0.41 (0.29–0.59)	0.000 0.000 0.030 NA NA	Zanrosso, 2012	[59]
	Brazil	ALL, AML	626/401	≤12	*NQO1, PON1*	*PON1*-55M in non-Whites *PON1*-55M in ALL >1–10 years *PON1*-192R in ALL >1–10 years *NQO1*-609T in AML ≤ 1 year *NQO1*-609T in ALL *MLL*- ≤12 months *NQO1*-609T in ALL *MLL*- >12–24 months	2.52 (1.49–4.26) 1.99 (1.17–3.39) 0.57 (0.33–0.97) 0.26 (0.10–0.68) 0.36 (0.16–0.81) 2.36 (1.02–5.72)	NA NA NA NA NA NA	De Aguiar Gonçalves, 2012	[39]
	Brazil	ALL	204/364	mean3.9	*CYP3A5, NAT2*	CYP3A5*3 (+/+) in White children CYP3A5*3 (–/+; +/+) in White children CYP3A5*6 (–/+; +/+) in White children CYP3A5*6 (–/+; +/+) in non-White children	0.38 (0.16–0.90) 0.43 (0.18–1.00) 3.80 (1.10–13.59) 0.32 (0.11–0.90)	0.030 0.050 0.020 0.050	Silveira, 2012	[37]

*med.:* median of age.

*MLL+*: positive for *MLL* gene rearrangement.

*MLL–*: negative for *MLL* gene rearrangement.

(–/+): heterozygote for the variant allele.

(+/+): homozygote for the variant allele.

AL: acute leukaemia.

ALL: acute lymphoblastic leukaemia.

AML: acute myeloid leukaemia.

OR: *odds ratio*. 95%CI: 95% confidence interval.

NA: not available.

a only lower bound of confidence interval was presented by the authors.

b relative risk (RR).

c ABCB1 (MDR1), ABCC1 (MRP1), ABCC2 (MRP2), AhR, ARNT, COMT, CYP1A1, CYP1A2, CYP1B1, CYP2A6, CYP2B6, CYP2C19, CYP2C8, CYP2D6, CYP2E1, CYP3A4, CYP3A5, EPHX1, EPHX2, FMO3, GCLC, GGT1, GPX6, GSR, GSS, GSTA1, GSTO2, GSTP1, IDH1, MPO, NAT1, NAT2, NQO1, PON1, PTGS1, PTGS2, SULT1A1, TPMT, UGT1A1, UGT1A7, UGT1A9, UGT2B7.

**Table 2.** Genetic susceptibility publications in childhood leukaemia involving genes related to DNA repair.

Continent	Country	Leukaemia subtype	Numberof cases/controls	Ages of cases(years)	Investigated genes	Significant results			First author, year	Reference
						Genetic variation	OR (95% CI)	P-value		
**Europe**	Poland	ALL	270/6984	mean6.65	*NBN*	*NBN* 657del5 carriers	1.85 (1.42–2.65)	0.035	Chrzanowska, 2006	[76]
	Poland	ALL	398/731	*med.* 4.9	*NBN*	*NBN* 657-wt/del5 *NBN* 657-del5/del5	3.01 (2.42–3.85) 1325.21 (859.84–2167.90)	0.004 0.003	Pastorczak, 2011	[77]
	Poland	ALL	97/131	mean5.4	*OGG1, MUTYH, XRCC1*	*OGG1* 326-Ser/Ser *OGG1* 326-Cys/Cys *OGG1* 326-Cys *XRCC1* 399-Arg/Arg + *OGG1* 326-Ser/Ser *XRCC1* 399-Arg/Gln + *OGG1* 326-Cys/Cys *OGG1* 326-Ser/Ser + *MUTYH* 165-Tyr/Tyr *OGG1* 326-Cys/Cys + *MUTYH* 165-Tyr/Tyr	0.45 (0.26–0.76) 5.36 (1.90–15.09) 2.33 (1.53–3.55) 0.40 (0.19–0.83) 3.83 (1.00–14.86) 0.43 (0.25–0.73) 6.75 (2.19–20.77)	0.003 0.001 <0.001 0.013 0.050 0.001 <0.001	Stanczyk, 2011	[74]
	Turkey	Pre-B ALL	52/60	mean5.9	*XRCC1, ERCC2*	No significant associations were found.	–	–	Celkan, 2008	[69]
	Turkey	ALL	70/75	≤15	*XRCC1, ERCC2*	*XRCC1* codon 194-Arg/Trp + Trp/Trp in girls	5.47 (1.49–20.10)	0.008	Batar, 2009	[67]
	Turkey	ALL	167/190	1.5–15.5	*XRCC1*(+ *CYP2E1*)	*XRCC1* codon 399-Arg/Gln +Gln/Gln *XRCC1* codon 399-Arg/Gln +Gln/Gln in girls *XRCC1* codon 399-Gln + CYP2E1*5B-*6 *XRCC1* codon 399-Gln +CYP2E1*5B-*6 in girls	1.60 (1.00–2.40) 2.10 (1.10–3.90) 3.70 (NA) 17.40 (1.90–153.70)	0.040 0.020 0.049 0.001	Tumer, 2010	[75]
**Asia**	India	ALL	117/117	≤14	*XRCC1*	*XRCC1* codon 399-Gln/Gln *XRCC1* codon 399-Arg/Gln *XRCC1* codons 194-Trp + 399Gln	2.42 (1.00–5.89) 1.90 (1.08–3.35) 4.41 (1.83–10.61)	0.050 0.030 0.009	Joseph, 2005	[71]
China	ALL	183/190	≤18	*ERCC1*	*ERCC1* 8092-CC *ERCC1* 8092-CC in boys *ERCC1* 19007-GG in boys *ERCC1* 8092-CC in children <8 years of age	1.61 (1.03–2.50) 1.94 (1.09–3.41) 2.36 (1.05–5.27) 1.87 (1.04–3.37)	0.030 0.020 0.040 0.040	Wang, 2006	[80]
	China	ALL	415/511	1–18	*OGG1*	*OGG1* 326-Ser/Ser +Ser/Cys	0.66 (0.49–0.88)	0.005	Li, 2011	[79]
	Thailand	ALL	108/317	≤14	*XRCC1,ERCC2*	*XRCC1* codon 194-Trp/Trp *XRCC1* codon 399-Arg/Gln +Gln/Gln *XRCC1* haplotype B(194Trp-280Arg-399Arg) *XRCC1* haplotype C(194Arg-280Arg-399Gln)	0.22 (0.05–0.96) 2.18 (1.39–3.42) 0.62 (0.42–0.90) 1.59 (1.14–2.23)	0.030 0.001 0.010 0.008	Pakakasama, 2007	[73]
	Taiwan	ALL, AML	266/266	<18	*XRCC4*	*XRCC4* rs6869366rs28360071: TT/DD *XRCC4* rs6869366rs28360071: GT/II *XRCC4* rs6869366rs28360071: GT/ID *XRCC4* rs6869366rs28360071: GT/DD	2.82 (1.03–7.70) 2.16 (1.29–3.61) 2.26 (1.22–4.17) 4.94 (1.01–24.27)	0.048 0.003 0.009 0.040	Wu, 2010	[81]
**North America**	Canada	ALL	287/320	*med.* 5	*MLH1, MSH3*(+ *GSTM1, CYP1A1, CYP2E1, NQO1, NAT2*)	*MLH1* 219-Ile/Ile + CYP2E1*5 (–/+, +/+) *MLH1* 219-Ile/Ile + *GSTM1*-null + CYP1A1*2A (–/+, +/+)	15.80 (2.00–122.60) 6.00 (1.90–18.90)	<0.001 0.002	Mathonnet, 2003	[78]
	Mexico(Hispanics)	ALL	120/120	≤14	*XRCC1*	*XRCC1* haplotype B(194Trp-280Arg-399Arg) *XRCC1* haplotype B(194Trp-280Arg-399Arg) in boys	1.95 (1.13–3.37) 2.65 (1.25–5.63)	0.016 0.010	Meza-Espinoza, 2009	[72]
	United States (Caucasians)	ALL	163/251	<21	*MLH1, MSH2, MSH3*	No significant associations were found.	–	–	Beuten, 2011	[21]
	United States (Hispanics/non-Hispanics)	ALL	335/490	mean 5.5 mean 5.6	Haplotypes of 32 genes(21 genes related to DNA repair systems^a^)	*ERCC2* (rs3916874,rs238416, rs171140) GAA *APEX1* (rs11160711, rs3120073) AA in non-Hispanics *BRCA2* (rs4942448,rs9943876) GA in non-Hispanics *RAD51* (rs2304579,rs7177265, rs2304580) AAA in Hispanics *RAD51* (rs2304579,rs7177265, rs2304580) AGA in Hispanics *NBN* (rs12680687, rs6470522, rs7840099, rs1805812,rs709816) rare haplotypes in ALL with t (12; 21) *XRCC4* (rs7711825, rs1193695, rs301276, rs301287, rs3777018)CGAGA in ALL with t (12; 21) *XRCC4* (rs7711825, rs1193695, rs301276, rs301287, rs3777018) CGGGA in ALL with t (12; 21) *XRCC4* (rs1193695, rs301276, rs301287) GAG in ALL with any structural change *XRCC4* (rs1193695, rs301276, rs301287) GGGin ALL with any structural change	0.59 (0.38–0.91) 1.90 (1.25–2.89) 1.77 (1.10–2.85) 1.55 (1.01–2.42) 1.51 (1.01–2.26) 0.38 (0.16–0.88) 0.56 (0.31–1.00) 0.39 (0.16–0.95) 0.60 (0.42–0.86) 0.55 (0.35–0.88)	0.018 0.003 0.020 0.050 0.040 0.025 0.050 0.039 0.006 0.012	Chokkalingam, 2011	[70]
**South America**	Brazil (Whites/non-Whites)	ALL	206/364	0.3–18	*XRCC1, ERCC2* (+ *TYMS*)	*TYMS* 2R/3R; 3R/3R + *XRCC1* 194-Arg/Arg + *XRCC1* 399-Arg/Gln; Gln/Gln *TYMS* 2R/3R; 3R/3R + *XRCC1* 194-Arg/Arg + *XRCC1* 399-Arg/Gln; Gln/Gln + *ERCC2* 751-Lys/Gln; Gln/Gln	0.25 (0.08–0.76) 0.25 (0.08–0.76)	0.005 0.005	Canalle, 2011	[68]

*med.:* median of age. ALL: acute lymphoblastic leukaemia. AML: acute myeloid leukaemia. OR: odds ratio. 95%CI: 95% confidence interval.

a *APEX1, MUTYH, UNG2, XRCC1, ERCC2, LIG4, PRKDC, XRCC4, XRCC5, XRCC6, BRCA1, BRCA2, MRE11, NBN, RAD50, RAD51, RAD54B, RAD54L, XRCC2, XRCC3* and *MGMT*.

**Table 3.** Genetic susceptibility publications in childhood leukaemia involving genes related to cell cycle regulation, signaling, proliferation and differentiation.

Continent	Country	Leukaemia subtype	Number of cases/controls	Age of cases(years)	Investigate dgenes	Significant results			First author, year	Reference
						Genetic variation	OR (95%CI)	P-value		
**Europe**	United Kingdom	ALL	114/414	≤14	*TP53*, *MDM2,* and others(*DAXX, BAT3, LTA, DDR1, IER3*)	*TP53* codon 72-Arg/Pro +Pro/Pro *BAT3* rs805303 *BAT3* rs2077102 *DAXX* rs2239839-rs1059231rs2073524	1.67 (1.21–2.30) 0.68 (0.49–0.95) 0.62 (0.39–0.99) 2.45 (1.22–4.91)	0.002 0.020 0.040 0.010	Do, 2009	[83]
	Germany/United Kingdom	Pre-B ALL	1384/1877	mean 6	*IKZF1, ARID5B, CEBPE*	*IKZF1* rs4132601-AC *IKZF1* rs4132601-CC *ARID5B* rs7089424-AC *ARID5B* rs7089424-CC *CEBPE* rs2239633-GG	1.80 (1.50–2.00) 2.80 (2.20–3.60) 1.80 (1.50–2.10) 3.20 (2.60–4.00) 1.60 (1.30–1.90)	<0.001 <0.001 <0.001 <0.001 <0.001	Prasad, 2010	[95]
	Poland	ALL	398/731	*med.* 4,9	*IKZF1, ARID5B, CEBPE, CDKN2A*	*IKZF1* rs4132601-G *ARID5B* rs7089424-G	1.34 (1.11–1.61) 1.33 (1.10–1.61)	0.002 0.003	Pastorczak, 2011	[77]
	Hungary	ALL	543/529	1–15	16 genes^a^	*ARID5B* rs10821936 in B-ALL *ARID5B* rs7089424 in B-ALL *ARID5B* rs4506592 in B-ALL *IKZF1* rs6964969 in B-ALL *IKZF1* rs11978267 in B-ALL *IKZF1* rs4132601 in B-ALL *STAT3* rs3816769 in hyperdiploid ALL *STAT3* rs12949918 in hyperdiploid ALL	1.53 (1.26–1.85) 1.52 (1.25–1.84) 1.51 (1.24–1.83) 1.70 (1.40–2.08) 1.68 (1.38–2.05) 1.69 (1.38–2.06) 0.62 (0.49–0.79) 0.64 (0.50–0.81)	<0.001 <0.001 <0.001 <0.001 <0.001 <0.001 <0.001 <0.001	Lautner-Csorba, 2012	[43]
**Asia**	Israel	T-ALL	39/200	NA	*ATMTP53*	*ATM*-T1229C, T1744C, T4388G *ATM*-C103T, -30del215, 2284delCT	4.90 (1.20–18.20) 12.90(2.50–42.70)	0.030 0.004	Liberzon, 2004	[84]
	China	ALL	183/190	mean 9.32	*CCND1*	*CCND1* 870AA versus. AG+GG	3.29 (1.99–9.02)	0.021	Hou, 2005	[82]
	China	ALL	570/673	1–18	*TERT*	*TERT* rs2735940-TT *TERT* rs2853676-AG *TERT* rs2736100-CC *TERT* rs10069690-AA *TERT* rs4246742-TA	1.38 (1.00–1.90) 1.36 (1.06–1.74) 1.56 (1.11–2.21) 2.00 (1.03–3.88) 0.78 (0.61–1.00)	0.034 0.010 0.006 0.032 0.029	Sheng, 2013	[85]
	Thailand	ALL	190/182	mean 6.0	*IKZF1, ARID5B, CEBPE, CDKN2A*	*IKZF1* rs4132601-C *ARID5B* rs10821938-C inpre-B ALL	1.57 (1.01–2.44) 0.73 (0.55–0.97)	0.040 0.030	Vijayakrishnan, 2010	[97]
**North America**	Canada	Pre-B ALL	240/277	0.4–18	*CDKN2A, CDKN2B, CDKN1A, CDKN1B*	*CDKN2A* -222-A *CDKN2A* -222-TA *CDKN2B* -593-T *CDKN2B* -1270/-593/-287CTG haplotype *CDKN2B* -1270/-593/-287CAG haplotype *CDKN1B* -1608-GA	2.20 (1.20–4.00) 2.60 (1.10–4.30) 0.70 (0.60–1.00) 0.80 (0.60–1.00) 1.70 (1.20–2.40) 1.70 (1.00–2.80)	0.008 0.010 0.020 0.040 0.004 0.030	Healy, 2007	[16]
	Canada	Pre-B ALL	284/270	*med.* 4.2	*ARID5B*	*ARID5B* rs7073837-AA *ARID5B* rs10994982-AA *ARID5B* rs10740055-CC *ARID5B* rs10821936-CC *ARID5B* rs7089424-CC *ARID5B*-AACCG haplotype	2.37 (1.45–3.85) 2.29 (1.42–3.69) 2.76 (1.68–4.53) 3.11 (1.90–5.10) 3.11 (1.89–5.12) 1.93 (1.47–2.53)	<0.001 <0.001 <0.001 <0.001 <0.001 <0.001	Healy, 2010	[94]
	United States (Caucasians)	AML	432/496	<21	*MDM2*	*MDM2* 309-GG	1.50 (1.03–2.20)	NA	Phillips, 2010	[86]
	United States (Caucasians)	ALL	163/251	<21	23 genes^b^	*LMO1* rs442264 *LMO1* rs442264 in B-ALL	1.90 (1.41–2.56) 1.98 (1.44–2.73)	<0.001 <0.001	Beuten, 2011	[21]
	United States (Hispanics/non-Hispanics)	B-ALL	203/414	mean6.8/6.0	*MLL, CREBBP, TOP2A,EP300*	*MLL* rs525549-AA versus AT/TT in Hispanics *MLL* rs6589664-GG versusAG/AA in Hispanics *MLL* rs6589664-AA versus AG/GG in Whites *EP300* rs5758222-GG/AG versus AA in Hispanics *EP300* rs7286979-AA/AG versus GG in Hispanics *EP300* rs20551-AA/AG versus GG in Hispanics *MLL-EP300* AG-GAA haplotype	2.56 (1.02–6.41) 2.49 (1.23–5.04) 0.39 (0.17–0.91) 2.79 (1.46–5.32) 2.67 (1.40–5.07) 2.79 (1.26–6.18) 5.68(2.82–11.44)	0.044 0.011 0.029 0.002 0.003 0.011 <0.001	Piwkham, 2011	[87]
	United States (Hispanics/non-Hispanics)	ALL	335/490	mean 5.5/5.6	Haplotypes of 32 genes(8 genes related to cell cycle and to poisomerase ^c^)	*CDKN2A* (rs3731257rs2518719) GG in hyperdiploid AL *CDKN2A* (rs3731257rs2518719) AA and GG in AL with any numerical ploidy change	0.30 (0.14–0.64) 0.67 (0.50–0.90) 0.44 (0.26–0.73)	0.0020.0080.001	Chokkalingam, 2011	[70]
	United States (Hispanics/non-Hispanics)	Pre-B ALL	1308/1587	<21	*ARID5B*	*ARID5B* rs10821936 in non-Hispanics *ARID5B* rs10821936 in Hispanics *ARID5B* rs7915732 in Hispanics	2.13 (1.77–2.58) 1.92 (1.50–2.45) 2.58 (1.27–3.52)	<0.001 <0.001 0.009	Xu, 2012	[98]
	United States (non-Hispanics)	ALL, AML	171/384	<1	*IKZF1, ARID5B, CEBPE*	*IKZF1* rs11978267-GG *ARID5B* rs10821936-C allele in ALL *MLL-* *ARID5B* rs10994982-A allele in AML *MLL+*	2.30 (1.30–4.20) 2.80 (1.60–5.00) 0.50 (0.30–0.90)	<0.050 <0.050 <0.050	Ross, 2013	[96]

*med.:* median of age.

*MLL+*: positive for *MLL* gene rearrangement.

*MLL–*: negative for *MLL* gene rearrangement.

AL: acute leukaemia.

ALL: acute lymphoblastic leukaemia.

AML: acute myeloid leukaemia.

OR: *odds ratio*. 95% CI: 95% confidence interval.

NA: not available.

a *ARID5B, BAX, BCL2A, BCL2B, CCR5, CEBPA, CEBPE, IKZF1, JAK1, JAK3, NOTCH1, STAT1, STAT3, STAT5A, STAT5B, STAT6*.

b *BCR, ABL1, ETV6, FBXW7, LMO1, LYL1, EP300, CREBBP, MLL, JAK2, RUNX1, TCF3, CHEK2, ATM, CCND1, TOP2A, CDKN1B, IKZF1, NR3C1, TP53, BLNK, CD6, SAMSN1*.

c *TP53, TP53BP1, CCND1, CDKN2A (p16), CDKN2B (p15), TOP1, TOP2A, TOP2B*.
